# EQ-5D-5L value set for Norway: a hybrid model using cTTO and DCE data

**DOI:** 10.1007/s11136-024-03837-3

**Published:** 2024-11-20

**Authors:** Andrew M. Garratt, Knut Stavem, James W. Shaw, Kim Rand

**Affiliations:** 1 Division for Health Services, Norwegian Institute of Public Health, Oslo, Norway; 2 Health Services Research Unit, Akershus University Hospital, Nordbyhagen, Norway; 3 Institute of Clinical Medicine, University of Oslo, Oslo, Norway; 4 Department of Pulmonary Medicine, Medical Division, Akershus University Hospital, Nordbyhagen, Norway; 5 Bristol Myers Squibb, Princeton, NJ USA

**Keywords:** EQ-5D-5L, General population, Outcomes, Quality of life, Utilities, Value set

## Abstract

**Purpose:**

To develop the Norwegian value set for the EQ-5D-5L based on interviews with a representative sample of the Norwegian adult population.

**Methods:**

Random and quota sampling were used to recruit the sample of adults (age> 18 years) representative of the Norwegian general population. Data collection followed EQ-VT 2.1 undertaken before and after the COVID-19 pandemic from November 2019 to December 2022, using PC-assisted and video conferencing interviews, respectively. Each respondent valued 10 health states using composite time trade-off (cTTO) and 7 health states using a discrete choice experiment (DCE). Different statistical models were assessed for logical consistency and predictive accuracy using cTTO and DCE data alone or in combination as hybrid models.

**Results:**

Of the 1,321 respondents, 1,237 met inclusion criteria. All statistical models demonstrated logical consistency. The weighted hybrid model combining both cTTOand DCE data was preferred and had the highest predictive accuracy. Predicted values ranged from -0.453 to 1, and the dimension of anxiety/depression was the most highly valued by respondents, followed by pain/discomfort, self-care, mobility, and usual activities. These findings are not dissimilar to those for most Western European countries, and regression coefficients are closest to those for other Scandinavian countries.

**Conclusion:**

This study provides the Norwegian value set for the EQ-5D-5L based on health state values obtained from members of the adult general population in Norway. This is an important contribution to economic evaluation and the broader application ofthe EQ-5D-5L in Norway including clinical and health services research, and quality measurement.

**Supplementary Information:**

The online version contains supplementary material available at 10.1007/s11136-024-03837-3.

## Introduction

Health economic evaluation undertaken by the Norwegian Institute of Public Health and the Norwegian Medical Products Agency includes the quality-adjusted life years (QALY) methodology and use of the EuroQol EQ-5D for assessing health outcomes [1, 2]. This follows similar recommendations for other countries, many of which have their own national value set for scoring the EQ-5D based on general population surveys [3, 4]. The EQ-5D is the most widely used patient-reported outcome measure (PROM) in economic evaluation and is used in research more generally, including in Scandinavian national medical registers [5, 6].

The most recent EQ-5D with five-levels (EQ-5D-5L) has a descriptive system which includes five dimensions of health: mobility, self-care, usual activities, pain/discomfort, and anxiety/depression. Each dimension has five levels reflecting no problems, slight problems, moderate problems, severe problems, and extreme problems/unable to do. The five responses give a health state represented by five-digits (for example, 12231), beginning with mobility. Health states are scored to give an index using a scoring algorithm from a value set derived from valuation tasks typically undertaken with general population samples [3]. These values are anchored in 1 (= full health) and 0 (= dead) with values < 0 indicating states valued less than dead. Values in the form of a scoring algorithm inform the economic evaluation of health technologies based on cost per QALY.

Further national value sets were developed following the introduction of the five-level version, the EQ-5D-5L, which now cover at least 38 countries [3, 4, 7–17]. These have largely followed the EuroQol Valuation Technology (EQ-VT) protocol, which provides methodological consistency, quality control, and promotes best practices and comparability across countries [3, 18]. The EQ-VT is available for computer/PC-assisted face-to-face and video conferencing interviews [8], which include the composite time trade-off (cTTO) and discrete choice experiment (DCE) valuation methods [18, 19].

EQ-5D-5L use is increasing in Norway, and it is the most widely used PROM in the national system of medical registers. Norway lacks a value set for the EQ-5D-5L, and national recommendations are use of an interim crosswalk, which maps the EQ-5D-5L to the original UK EQ-5D-3L value set [2, 20, 21]. However, the crosswalk is based on the UK value set developed in the 1990s for the earlier EQ-5D descriptive system, and before important methodological advances including EQ-VT. The availability of a Norwegian value set will increase the legitimacy of EQ-5D-5L use across applications and particularly economic evaluation. This study followed the EQ-VT protocol [18, 19] to derive a Norwegian value set for the EQ-5D-5L based on interviews with a representative sample of the adult Norwegian general population.

## Methods

The study followed the latest protocol from the EuroQol Group (EQ-VT 2.1) [18], and reporting follows the CREATE checklist [22]. Data collection started in November 2019 using face-to-face interviews [19] but was postponed in March 2019 due to the COVID-19 pandemic. Data collection resumed with face-to face and video conferencing interviews in the final two months of 2022.

### Ethics

The Regional Committee for Medical and Research Ethics stated that the study did not require their approval. The Data Protection Impact Assessment was approved by the Norwegian Institute of Public Health on 30 September 2019. The continuation of data collection in 2022 was assessed by the Data Protection Officer at Akershus University Hospital, who concluded that the impact assessment of 2019 was still valid.

### Sample selection and data collection

Respondents were aged 18 years and over, resided in Norway and were sufficiently literate in Norwegian to complete the interview. Sample size was set to a minimum of 1,000 individuals with each valuing 10 health states, with the aim of 10,000 responses [3, 23]. Multistage random sampling and quota sampling were used to ensure representativeness according to age, sex, educational level, and geography, as described in the protocol [19]. Five hospital areas were randomly selected such that a minimum of one would be in each of the Norwegian health regions (North, East/South, Middle, West), with sampling likelihood weighted by the average number of individuals in the catchment area. Within these samples, quota sampling was used to achieve national representativeness [19]. Hard to reach groups were oversampled including ethnic minorities, those with lower socio-economic status, and parents of young children. Potential respondents were informed about a cash card incentive equivalent to €30 at interview completion. Data collection took place at different locations [19].

For the video conferencing and to ensure accordance with quotas, interviewers completed an interview scheduling tool with available slots filled by recruiters. Recruiters were awarded a fixed hourly rate and based on previous experiences of recruitment challenges, more time was allotted for participants who were male, aged over 65 years, and with lower educational level. Participants received a reminder prior to the scheduled time and an interview link. The interviewer shared the screen with the respondent and managed data entry [8].

### Valuation interviews

Interviewers were graduate students at the University of Oslo, except 2 who were retired healthcare professionals. All underwent 2.5 days of intensive training and undertook 10 test interviews with approved quality according to the EQ-VT protocol [24] before main data collection.

The interviewer guided the respondent throughout and answered any questions. Their instructions included not commenting on possible illogical responses, encouraging thinking aloud, and asking respondents to carefully consider each health state. Interview content was identical for the two data collections. For the face-to-face interviews, the portable version of the software (EQ-PVT) was used, which has the same functionality and interface as the standard web-based version 2.1. The interviews have five sections. First, introduction and welcoming the respondent. Second, respondents completed the EQ-5D-5L and questions about age, gender (male, female, other), and education level. Third, the cTTO was introduced including an explanation of the task and completion of three practice tasks for mild, moderate, and severe health states. Respondents were randomized to 1 of 10 standardized cTTO blocks of EQ-5D-5L health states, each of which included one very mild state (11112, 11121, 11211, 12111, or 21111), the worst state (55555), 8 states of different severity, and covering a total of 86 health states [23, 24]. Following completion of the cTTO, respondents were presented with a feedback module comprising the 10 health states in order of the values they assigned, and asked to flag responses that were clearly misordered. Fourth, the DCE was introduced along with instructions. Respondents were randomized to 1 of 28 standardized blocks of 7 DCE pairs [23, 24]. Fifth, respondents were thanked for their participation.

cTTO valuation starts with the standard time trade-off (TTO), applicable to health states valued higher than dead, and changes to the lead-time TTO for states valued lower than dead by the respondent. Values range from − 1 to 1 for the lowest (trading all lead time) and highest (trading no time) valued health state with 0.05 increments. DCE asks respondents to choose between two EQ-5D-5L health states in terms of preference.

QC was used at weekly intervals to begin with, and then at 2 to 4-week intervals to monitor interviewer protocol compliance and face validity of cTTO data. Flags included time spent on the task, introduction to lead-time TTO during the initial example of the health state, and inconsistent valuations of the worst possible health state [20].

### Modelling and data analysis

Descriptive statistics were used to compare characteristics of respondents with those for the Norwegian general population (Statistics Norway, October 1, 2022). Prior to statistical modelling, cTTO data for health states flagged in the feedback module were removed along with cTTO and DCE data not complying with the study protocol.

Respondent values for the 86 EQ-5D-5L health states were used to estimate the 3,125 possible health state values. Modelling was undertaken for cTTO data alone and combined with DCE data for the hybrid model [3, 4], using the widely used 20-parameter (Eq. 1) and 8-parameter (Eq. 2) models [7, 25]. The models have the same dependent and independent variables but differ by estimated coefficients. Following convention, the dependent variable was rescaled to disutilities (1-cTTO). The models were tested with and without intercepts, for a total of 8 tested combinations: cTTO alone vs. cTTO + DCE hybrid; 8 or 20 parameter form; with and without an intercept.

Let *l* be level ∈ {2,3,4,5}, *d* be dimension ∈ {MO, SC, UA, PD, AD}, and *x* be a vector of dummies.

Equation 1, 20-parameter model with intercept α:

$$\:-\left(u-1\right)=\:\alpha\:+$$

$$\:{{\beta\:}_{MO2}x}_{MO2}+{{\beta\:}_{SC2}x}_{SC2}+{{\beta\:}_{UA2}x}_{UA2}+{{\beta\:}_{PD2}x}_{PD2}+{{\beta\:}_{AD2}x}_{AD2}+$$

$$\:{{\beta\:}_{MO3}x}_{MO3}+{{\beta\:}_{SC3}x}_{SC3}+{{\beta\:}_{UA3}x}_{UA3}+{{\beta\:}_{PD3}x}_{PD3}+{{\beta\:}_{AD3}x}_{AD3}+$$

$$\:{{\beta\:}_{MO4}x}_{MO4}+{{\beta\:}_{SC4}x}_{SC4}+{{\beta\:}_{UA4}x}_{UA4}+{{\beta\:}_{PD4}x}_{PD4}+{{\beta\:}_{AD4}x}_{AD4}+$$

$$\:{{\:\:\:\beta\:}_{MO5}x}_{MO5}+{{\beta\:}_{SC5}x}_{SC5}+{{\beta\:}_{UA5}x}_{UA5}+{{\beta\:}_{PD5}x}_{PD5}+{{\beta\:}_{AD5}x}_{AD5}+\:\epsilon\:$$

Equation 2, 8-parameter model with intercept α:

$$\:-\left(u-1\right)=\alpha\:+$$

$$\:\left({{\beta\:}_{MO}x}_{MO2}+{{\beta\:}_{SC}x}_{SC2}+{{\beta\:}_{UA}x}_{UA2}+{{\beta\:}_{PD}x}_{PD2}+{{\beta\:}_{AD}x}_{AD2}\right){L}_{2}+$$

$$\:\left({{\beta\:}_{MO}x}_{MO3}+{{\beta\:}_{SC}x}_{SC3}+{{\beta\:}_{UA}x}_{UA3}+{{\beta\:}_{PD}x}_{PD3}+{{\beta\:}_{AD}x}_{AD3}\right){L}_{3}+$$

$$\:\left({{\beta\:}_{MO}x}_{MO4}+{{\beta\:}_{SC}x}_{SC4}+{{\beta\:}_{UA}x}_{UA4}+{{\beta\:}_{PD}x}_{PD4}+{{\beta\:}_{AD}x}_{AD4}\right){L}_{4}+$$

$$\:{{\beta\:}_{MO}x}_{MO5}+{{\beta\:}_{SC}x}_{SC5}+{{\beta\:}_{UA}x}_{UA5}+{{\beta\:}_{PD}x}_{PD5}+{{\beta\:}_{AD}x}_{AD5}+e$$

The hybrid model [26] combines cTTO and DCE data to give common coefficients found to be logically consistent and is widely used alongside EQ-VT versions 2.0 and 2.1 [3, 4]. This model uses joint maximum likelihood with the same parameters to give likelihood estimates over cTTO data assuming a normal distribution and DCE using conditional logit, applying the same set of coefficients to both, with an arbitrary parameter θ to account for the difference in scale between cTTO and DCE. In common with existing value sets [3, 4], potential models had censoring at -1 (right-censoring at disutility 2), random intercepts for cTTO at the individual respondent level, and allowed for heteroscedasticity linear in estimated state disutility, i.e. relaxing the homoscedasticity assumption by modeling the standard deviation of the fitted normal distribution for the model error, typically modeled as a single parameter σ, by $\:{\sigma\:}_{s}={\alpha\:}_{\sigma\:}+{\beta\:}_{\sigma\:}\times\:{v}_{s}$, where $\:{v}_{s}$ is the estimated disutility of EQ-5D-5L health states.

Final model selection was based on logical consistency in terms of ordering of EQ-5D-5L states and corresponding values, and out-of-sample predictive accuracy for TTO blocks as assessed by root mean square error (RMSE) between predicted health state values and likelihood-based (censored) mean values for the corresponding health states. Censored mean values were used to account for censoring at -1 and estimated using Tobit models to predict the mean value for each health state with a single coefficient per health state. Confidence intervals and standard errors for coefficients and all 3,125 predicted EQ-5D-5L health state values were derived using bootstrapping: 10,000 samples of the same size were drawn at the level of individual study participants, with resampling, the models were fitted to each subsample. Standard errors were estimated using the standard deviation of estimated coefficients and predicted health state values, and 95% confidence intervals were taken as the 2.5 and 97.5 percentiles of the bootstrapped values. To ensure representativeness of the sample and reduce potential biases in the analysis, the sample was re-weighted to match the general Norwegian population in terms of age, sex, and geographic region using propensity score weighting [27].

The distribution of the values in the new Norwegian value set were compared with values calculated using the crosswalk to UK 3 L values and EQ-5D-5L value sets from Denmark [28], Sweden [11], and US [29]. This was done by graphical display of key characteristics. Comparative value set sensitivity to change was also assessed graphically [30].

Models were fitted and tested in R 3.6.1 (R Development Core Team, Vienna, Austria).

## Results

### Sample characteristics

Protocol compliance as assessed by QC, was excellent across both data collections. Flags for the 1,321 respondents mostly related to less than five minutes completing the ten cTTO tasks (*n* = 28) and inconsistent valuations of the worst possible health state, or a value of 0.5 or above any other health state (*n* = 24). For the 1,237 respondents meeting inclusion criteria, 12,370 cTTO valuations and 8,659 DCE responses were included in the modelling. Each of the 1,237 respondents valued seven choice pairs resulting in 7,098 observations. Respondents were similar to the adult Norwegian general population (Statistics Norway, October 1, 2022) for age and gender (Table 1). Underrepresentation of > 5% compared to the general population included those with below secondary education, those with higher education of under four years, and those domiciled in the Southern region.

**Table 1:** Characteristic of respondents meeting the inclusion criteria (*n* = 1,237)

	Respondents		General Population^a^	
	n	%		%
Sex				
Female	647	52.3		49.8
Male	578	46.7		50.2
Other	12	1.0	-	-
Age				
18 to 34 years	340	27.5		28.5
35 to 66 years	649	52.5		51.8
67 years and over	248	20.0		19.7
Education				
Below upper secondary school	130	10.5		20.0
Upper secondary school	619	50.0		42.1
Higher education < 4 years	206	16.7		26.0
Higher education ≥ 4 years	273	22.3		11.9
Region				
East	632	51.1		37.6
Mid	154	12.4		13.7
North	103	8.3		9.2
South	135	8.3		19.0
West	240	19.4		20.5

^a^ Statistics Norway (http://www.ssb.no/befolkning) data for, October 1st, 2022

### Selection of the Norwegian EQ-5D-5L model

**Fig. 1:**
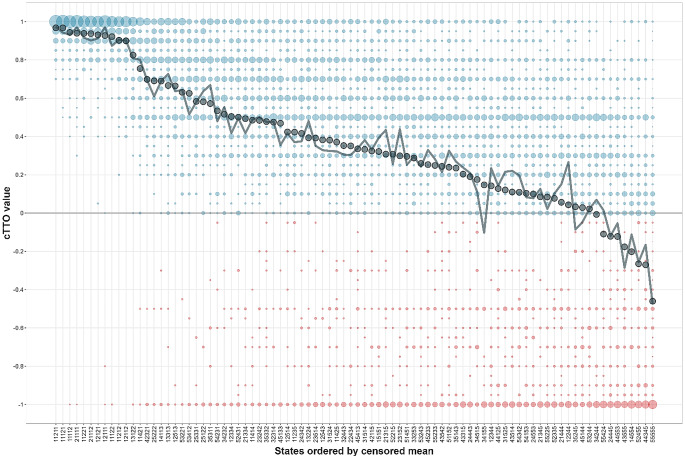
All assigned cTTO values, censored means, and predicted values for the 86 EQ-5D-5L health states included in the cTTO

**Fig. 2:**
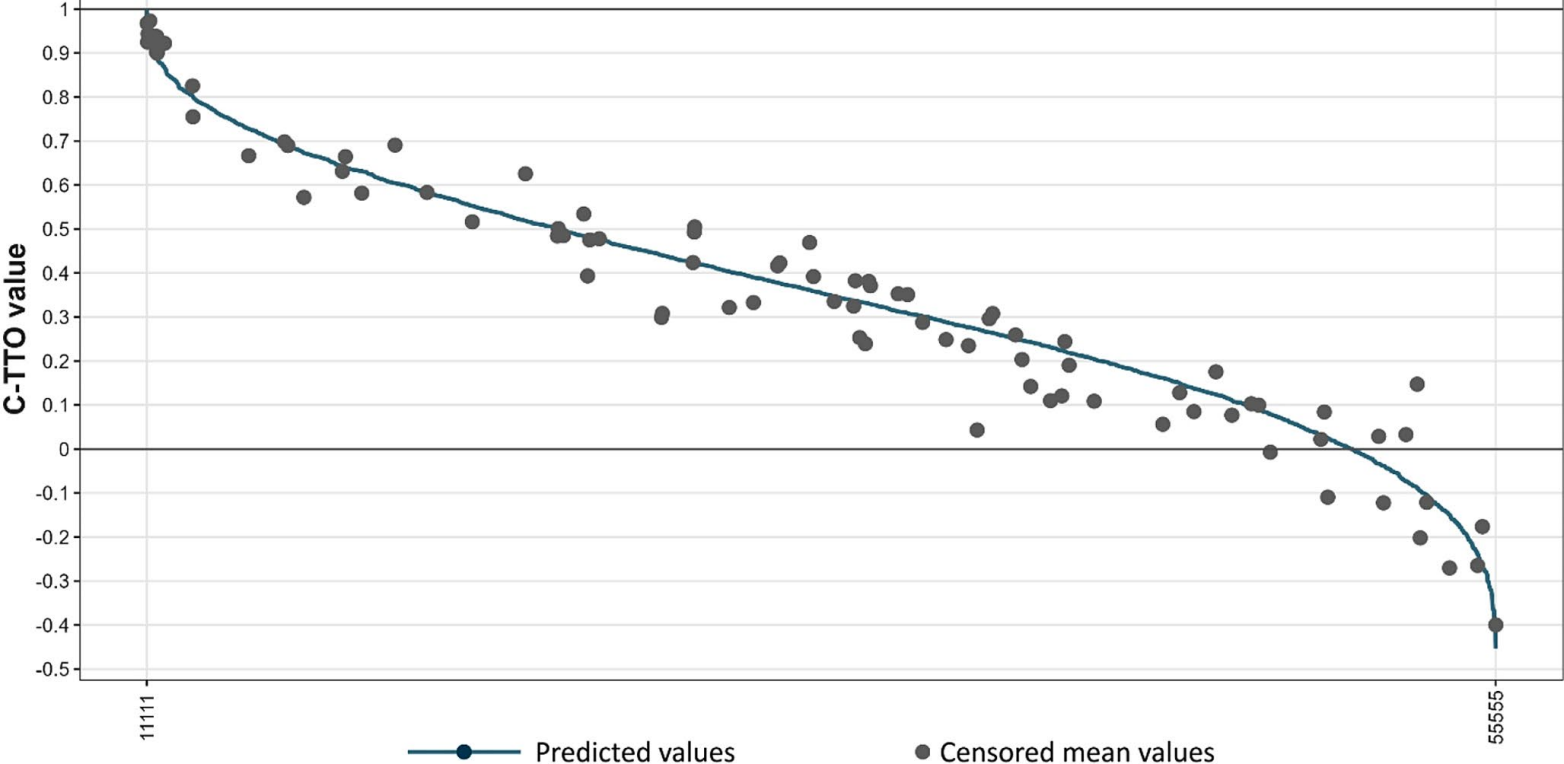
Predicted values for all 3125 EQ-5D-5L health states and censored means for the 86 directly valued cTTO health states

All models showed logical consistency with a larger decrease in estimated values for more severe health problems. Compared to the additive models, multiplicative models showed slightly greater predictive accuracy for the cTTO as assessed by the RMSE, including within hybrid models. Table 2 shows results for the two best performing cTTO models alongside respective hybrid models, which had slightly better predictive accuracy. The final Norwegian EQ-5D-5L value set was based on the hybrid model with no intercept (Table 2). Predictive values for the 3,125 health states along with SEs and 95% CIs are shown in the Supplementary File 1. Figure 1 shows the distribution of elicited cTTO values, the censored means, and predicted values for the 86 health states included in the cTTO. Figure 2 displays the range of the predicted values for all 3,125 EQ-5D-5L health states along with the censored means for the directly valued cTTO health states.

**Table 2:** Parameter estimates and predictive accuracy for final models^a^ (*n* = 1,237)

	cTTO hetero intercept		cTTO hetero		Hybrid intercept		Hybrid (Selected model)	
	Coefficient	SE^b^	Coefficient	SE	Coefficient	SE	Coefficient	SE
MO^c^	0.194	0.0120	0.199	0.0121	0.202	0.0083	0.205	0.0082
SC	0.200	0.0163	0.205	0.0159	0.201	0.0101	0.206	0.0096
UA	0.140	0.0158	0.154	0.0150	0.175	0.0085	0.179	0.0083
PD	0.394	0.0174	0.397	0.0175	0.388	0.0125	0.391	0.0123
AD	0.460	0.0193	0.473	0.0194	0.465	0.0134	0.472	0.0133
L2	0.084	0.0185	0.132	0.0204	0.135	0.0160	0.152	0.0166
L3	0.270	0.0278	0.294	0.0242	0.310	0.0140	0.317	0.0134
L4	0.829	0.0234	0.830	0.0227	0.771	0.0120	0.775	0.0119
Intercept	0.063	0.0174			0.036	0.0141		
RMSE^d^		0.0770		0.0778		0.0764		0.0764
MAE		0.0600		0.0591		0.0600		0.0578
Lin CCC		0.9821		0.9819		0.9823		0.9825
ICC		0.9821		0.9819		0.9823		0.9825
Pearson R		0.9827		0.9830		0.9830		0.9830

^a^ cTTO multiplicative model, DCE conditional probability model. Hybrid 8-parameter model without intercept. Random intercept at the individual level and linear heteroskedasticity. All models are weighted to be representative of the Norwegian general population

^b^ Standard errors based on bootstrapping with 10,000 resamples at the individual respondent level

^c^ Dimensions: MO mobility, SC self-care, UA usual activities, PD pain/discomfort, AD anxiety depression

^d^ RMSE root mean square error, MAE mean absolute error, CCC concordance correlation coefficient, ICC intraclass correlation coefficient, Pearson R correlation coefficient

For reasons of representatives the Norwegian value set was re-weighted to match the general population in terms of age, sex, highest attained educational level, and geographic region. Propensity score weighting for characteristics of the general population made very little difference to the results (Supplementary Files 1 and 2). The index score is calculated as follows:

$$\begin{aligned}U\left(15432\right)&=1-\left(\begin{aligned}&MO\cdot\:0+SC\cdot\:1+UA\cdot\:\:L4\cr&\quad+PD\cdot\:L3+AD\cdot\:L2\end{aligned}\right)\\&=1-\:\left(\begin{aligned}&0.205\cdot\:0+0.206\cdot\:1\cr&\quad+0.179\cdot\:0.775+0.391\cdot\:0.317\cr&\quad+0.472\cdot\:0.152\end{aligned}\right)=\:0.460\end{aligned}$$

**Fig. 3:**
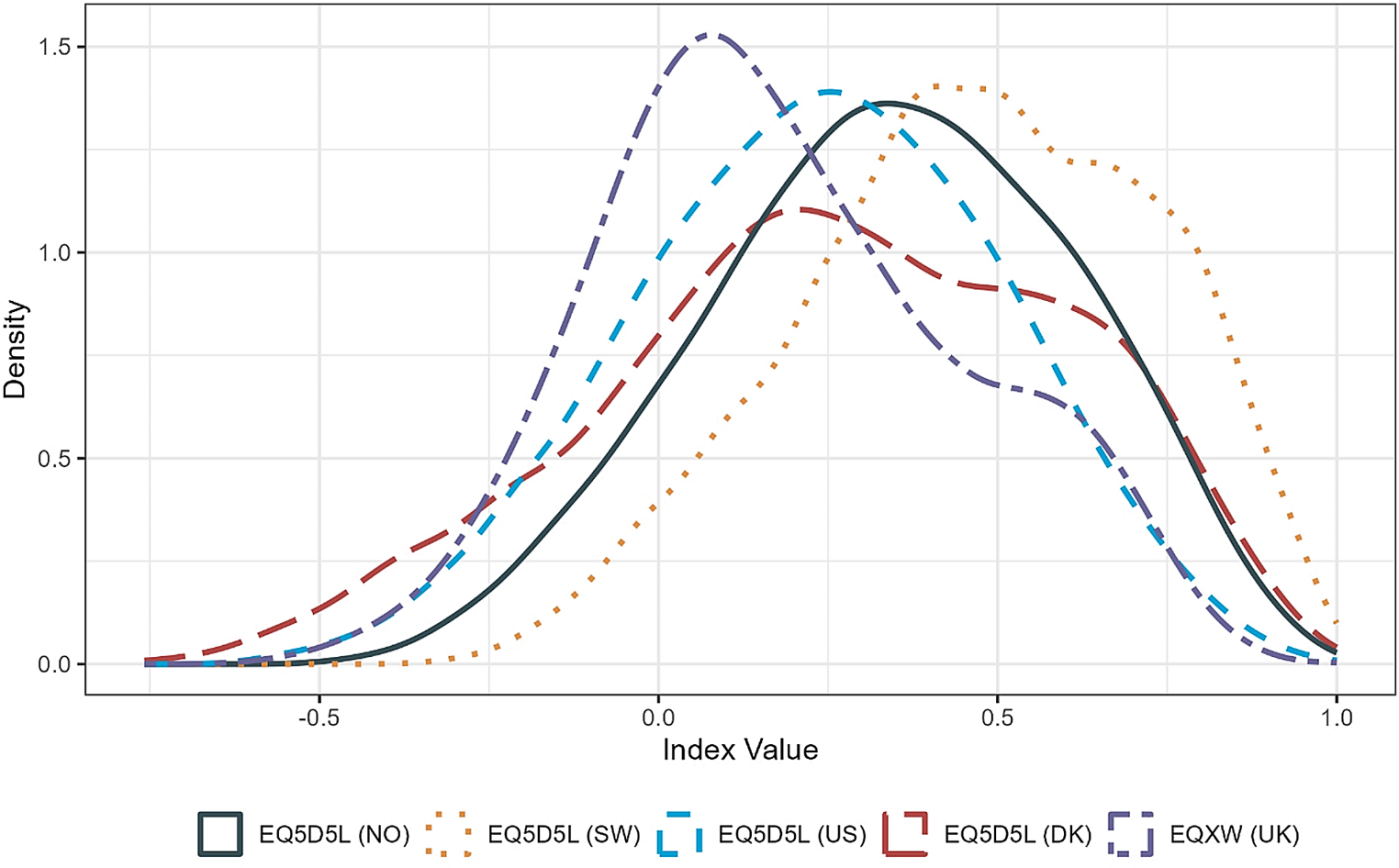
Density plots for the 3,125 EQ-5D-5L values predicted by the value sets for Norway (NO), UK crosswalk (UK), Denmark (DK), Sweden (SE), and United States (US)

The Norwegian value set has a lowest possible score of -0.453, and regression coefficients show that the dimensions of pain/discomfort and anxiety/depression (Table 2) make the largest contribution to health state values. Table 3 compares the score characteristics with those for the current crosswalk for Norway, other Scandinavian countries, and US. The distribution of Norwegian values is unimodal and close to symmetric (Fig. 3). Compared to the crosswalk there is a shift to higher values to the right of the distribution. The range of Norwegian values is within those for the crosswalk, Denmark and US but has a lower minimum value compared to Sweden (Table 3). Approximately 11% of health states are valued worse than dead compared to 20% for the crosswalk and others, except Sweden with 4%. For the crosswalk value set, mobility makes a relatively greater contribution than the other dimensions. Both anxiety/depression and pain/discomfort make the largest contributions for Denmark and Sweden. For the US, anxiety/depression is ranked fourth, with self-care ranked relatively higher.

**Table 3:** Key characteristics of Scandinavian, crosswalk, and US value sets

	Norway	Crosswalk (UK)	Denmark	Sweden	United States
Range of values	-0.453 to 1	-0.594 to 1	-0.758 to 1	-0.314 to 1	-0.573 to 1
Difference between best and next best state	0.027	0.094	0.033	0.010	0.057
States worse than dead (%)	333 (10.7)	834 (26.7)	684 (21.9)	147 (4.7)	624 (20.0)
^a^Mean (SD) single level transition across states	0.072 (0.013)	0.082 (0.020)	0.087 (0.022)	0.065 (0.020)	0.078 (0.014)
Number of participants	1,237	2,997	1,014	785	1,062
Model type	Weighted Censored Heteroscedastic 8 parameter TTO + DCE hybrid	10 parameter Non-censored Homoscedastic TTO only EQ-5D-3L crosswalk	Unweighted Censored Heteroscedastic 20-parameter TTO + DCE hybrid	Unweighted Censored Heteroscedastic 20-parameter TTO only	Unweighted Censored 20-parameter TTO only
^b^Dimension ranking	AD PD SC MO UA	PD MO AD SC UA	AD PD MO SC UA	PD AD UA SC MO	PD SC MO AD UA

^a^Mean single level transition across health states is the average of one move i.e. 3,124 states to the next

^b^Dimensions: MO mobility, SC self-care, UA usual activities, PD pain/discomfort, AD anxiety/depression

In the sensitivity plots for simulated EQ-5D-5L index values across the range of the EQ VAS, the Norwegian curve does not intersect the others but is closely parallel to that for Denmark, indicating similar sensitivity across a wide range of severity (Supplementary Fig. 1).

## Discussion

This study reports the Norwegian EQ-5D-5L value set based on surveys of the adult Norwegian general population using cTTO and DCE as part of the most recent EQ-VT 2.1 protocol to secure data quality. The value set gives the index score based on a scoring algorithm applied to responses and is suitable for estimating QALYs in economic evaluation. Application of a national value set improves the legitimacy of economic evaluation in Norway and is a major improvement over current recommendations for the interim crosswalk value set across research and other applications [2, 6, 31].

The crosswalk is based on the UK value set, derived three decades ago for the earlier EQ-5D descriptive system with three levels for each dimension. Methodological flaws are widely documented [3, 18, 21, 23, 24], which led to the development of the EQ-VT to address these deficiencies and provide greater standardization across nations to aid value set comparisons. There is increasing application of the 5 L version, and it is now the most widely used PROM in Norwegian national quality registers [6]. The availability of the value set and accompanying norm data [31] for the Norwegian general population is timely in this regard and enhances use of the five-level descriptive system.

The relative importance of dimensions across 31 EQ-5D-5L value sets was compared in a recent systematic review [4]. For standard hypothetical health state valuation studies, the results for Norway, with anxiety/depression and pain/discomfort as the two most important dimensions, follow those of 9 of 11 Western European countries including value sets more recently published [3, 4, 7, 8, 11]. Anxiety/depression was the most important for three of these countries. The dimension of mobility was the next most important after pain/discomfort in value sets for France, Portugal, and Italy [3, 4, 8]. More recent value sets for Eastern European countries found mobility [10, 14] and self-care [14] to be among the two most important dimensions, which together with anxiety/depression being one of the two least important dimensions, are similar for other Eastern European countries [3, 4].

When the size of regression coefficients is compared across dimension levels, those for Norway are within the range of those for Western European countries [3, 4, 7, 8, 11]. The exception is usual activities, the least important dimension in the Norwegian value set, which has slightly lower coefficients across three levels compared to Western European countries. When compared to those for other Western European countries, coefficients for the three Scandinavian countries [3, 4, 11, 28] are also closer in size. This is particularly true for usual activities, with the caveat that there is less variation for this dimension more generally. The lowest possible score of -0.453 is similar to the Netherlands [32] and Spain [33] and ranked fifth among 12 Western European countries. The number of states worse than dead at 11%, is closest to that for Portugal [34] and Spain [33] with 9 and 8%, respectively [3].

The comparison of EQ-5D-5L value sets is facilitated by the application of EQ-VT [3, 4]. The standardized protocol lends uniformity to valuation, promotes best practices for data collection, and includes QC procedures. Furthermore, the great majority of EQ-5D-5L value sets are based on either the cTTO or cTTO combined with DCE, with similar statistical modelling [3, 4], a possible synergy effect arising from EQ-VT use and greater awareness of good scientific practice. Hence, accruing evidence for differences across value sets is likely due to differences in values for health states associated with culture, income and wealth, and health systems rather than methods used to obtain EQ-5D-5L values [3, 4]. Research culture including attitudes and expectations about the way research is communicated and conducted, is also potentially important and might affect valuation studies differently across countries, including the interaction between interviewers and respondents.

The differences, including the relative importance of the dimensions and number of states worse than dead, lend support to the validity of national value sets but limit cross-national relevance and application in other countries. The uniqueness of the Norwegian value set including dimension rankings, lends further support for the use of country-specific value sets in economic evaluation.

The Norwegian value set followed the most recent version of the EQ-VT, version 2.1 to give high levels of data quality. This version added the feedback module as a further means of improving data quality. The Norwegian interviewers had substantial training, including EQ-VT presentations by the research team, a training workshop, detailed discussion of the interview guide, demonstration of EQ-PVT in an interview setting, pre-pilot interviews via role-playing, and feedback opportunities. Ten pilot interviews were subsequently conducted by all interviewers with feedback opportunities and QC in collaboration with the EuroQol Group. Members of the project team worked as interviewers including the Principal Investigator.

After comparing widely used models against standard criteria, the hybrid multiplicative 8-coefficient model without an intercept for cTTO data, random effects, and correction for heteroskedasticity was selected for the Norwegian value set data. This followed other national value sets where the combination of valuation data was informed by both sufficient agreement between cTTO and DCE and improvement in fit of observed and predicted values [3]. The hybrid model is the most popular and selected for the final value set in 19 of 27 studies using EQ-VT protocol 2.0 or 2.1 [3, 4, 7–12, 14–17].

The Norwegian EQ-5D-5L value set is considerably different to that currently recommended for Norway based on the crosswalk. The Norwegian values are higher which follows findings for several other countries where comparisons with EQ-5D-3L values and crosswalk were undertaken [29, 35–38]. Moreover, the ranking of dimensions has changed with anxiety/depression being most important compared to third most important in the crosswalk. The second largest change relates to mobility, which is the fourth most important compared to second most important in the crosswalk. Changes in dimension rankings were also found for France in comparisons of EQ-5D-3L and 5L value sets [35].

The EQ-VT protocol makes no requirements regarding sampling or representativeness, and several approaches were used across different countries. Norway has a low population density with remote areas, and the sampling strategy reflected this [19]. Multistage random and quota sampling were used to give representativeness in terms of age, sex, and geography. Moreover, locations were selected in a manner that would fulfill necessary quotas and hard to reach respondents including those in low socioeconomic groups and with time constraints. There was a lack of representativeness, with underrepresentation of respondents from the Southern region and a lower education level. It was decided to include weighting for the characteristics of the general population in the final value set. Only slight differences were found between the weighted and unweighted value sets in terms of performance across the different models tested.

Most EQ-5D-5L value set studies that considered representativeness through comparisons of respondent characteristics with those for the general population, found differences of > 5% for the background characteristics reported. This includes overrepresentation of more highly educated respondents and those from urban areas [3]. The two other Scandinavian countries of Denmark and Sweden found underrepresentation for age groups 18–24 and 30–49 years respectively [11, 28]. Denmark compared education levels and like Norway, found overrepresentation of the more highly educated [28]. The inclusion of weights was tested in models for five countries [3, 37–39] and included in the final value sets for England, France, and Peru [35, 38, 40].

The main study limitation arises from the COVID-19 pandemic preventing completion of the original data collection. The remaining half of the PC-assisted face-to-face interviews were due to take place from March to June 2020. Similar to Sweden [11], video conferencing was used following a delay of 2½ years. The time lag and the COVID-19 pandemic might have affected valuations but during this time, EQ-VT video conferencing became available with evidence for feasibility of data collection [8, 11]. Both data collections used the same methods of recruitment including use of contact persons at locations, and information materials. The revised protocol followed the original as far as possible and did not include consideration of mode of administration or possible interviewer effects arising from the recruitment of new interviewers. Both data collections adhered to EQ-VT 2.1 protocol and stringent criteria relating to training of interviewers, interviewer testing, and QC. Given the different interview methods, there are possibly differences in values for the two data collections. Both data collections had high levels of protocol compliance, and the decision was made not to test for this in the revised protocol.

## Conclusion

This study derived the Norwegian EQ-5D-5L value set based on the values of the Norwegian general population in 2019 and 2022. This value set should replace the currently recommended one based on crosswalk and the original UK EQ-5D-3L value set for the UK from 1996. This will serve to enhance the legitimacy of the Norwegian EQ-5D-5L in economic evaluation and other applications including clinical and health services research, and as a quality indicator in the national system of medical registers. The use of the standardized EQ-VT protocol will contribute to comparisons with existing and new value sets in an international context.

## Electronic supplementary material

Below is the link to the electronic supplementary material.

Supplementary Material 1

## Data Availability

Anonymized raw data is available at https://github.com/MathsInHealth/ValuationStudyData/.
