# Supplementary material for: EQ-5D-5L value set for Norway: a hybrid model using cTTO and DCE data
Source: Qual Life Res. 2024 Nov 20;34(2):417–27. doi: 10.1007/s11136-024-03837-3 (PMC11865167; doi:10.1007/s11136-024-03837-3)
Supplement: Supplementary file 1 — Supplementary Material 1 [file 11136_2024_3837_MOESM1_ESM.docx]

**Supplementary Figure 1**

Sensitivity plots where the solid line represents the mean index value for simulated populations derived around each EQ VAS score (0-100), standard error range by shaded areas and 95% confidence interval by dotted lines. Norway (NO), crosswalk (UKXW), Denmark (DK), Sweden (SE), and United States (US)


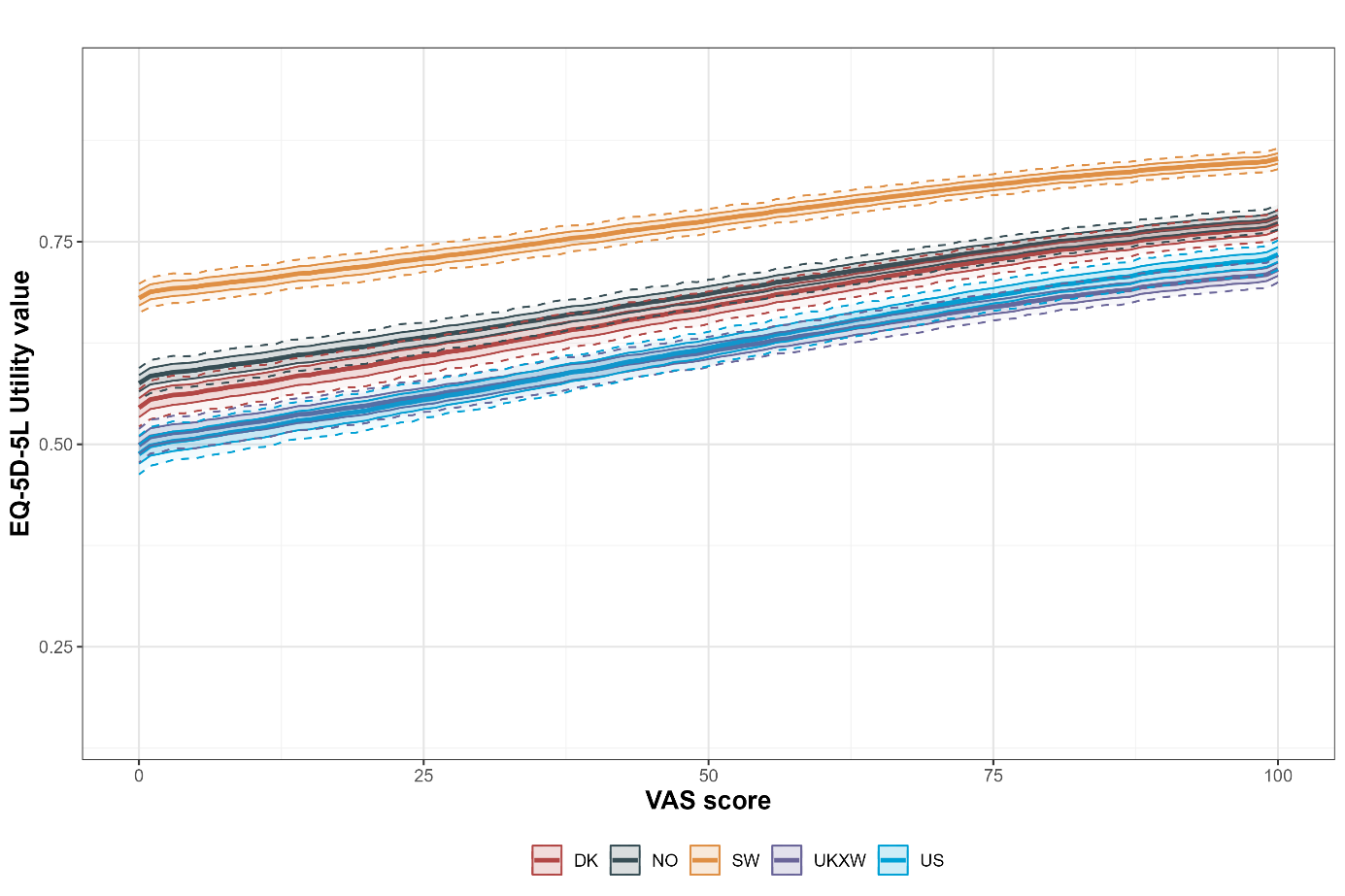


Article title: EQ-5D-5L value set for Norway: a hybrid model using cTTO and DCE data

Journal: Quality of Life Research

Authors: Andrew Garratt, Knut Stavem, James W Shaw, Kim Rand.

Corresponding author email: andrew.garratt@fhi.no
